# Type 1 Human Immunodeficiency Virus (HIV-1) Incidence, Adherence, and Drug Resistance in Individuals Taking Daily Emtricitabine/Tenofovir Disoproxil Fumarate for HIV-1 Pre-exposure Prophylaxis: Pooled Analysis From 72 Global Studies

**DOI:** 10.1093/cid/ciae143

**Published:** 2024-11-22

**Authors:** Raphael J. Landovitz, Li Tao, Juan Yang, Melanie de Boer, Christoph Carter, Moupali Das, Jared M. Baeten, Albert Liu, Karen W. Hoover, Connie Celum, Beatriz Grinsztejn, Sheldon Morris, Darrell P. Wheeler, Kenneth H. Mayer, Sarit A. Golub, Linda-Gail Bekker, Souleymane Diabaté, Elske Hoornenborg, Janet Myers, Ashley A. Leech, Sheena McCormack, Philip A. Chan, Michael Sweat, Lynn T. Matthews, Robert Grant, Global F/TDF PrEP Study Team, NZPrEP Demonstration Project, Chris Beyrer, Joelle Brown, Jesse Clark, Paul Colson, Robyn Eakle, Jason Farley, Charlene A. Flash, Jorge Gallardo, Geoffrey Gottlieb, Alexandre Grangeiro, Renee Heffron, Sybil Hosek, Mark Hull, John Idoko, Irene Inwani, Helen Koenig, Ann Kurth, Shui-shan Lee, Kenneth Mayer, Souleymane Mboup, Jaimie Meyer, Anthony Mills, Andrew Mujugira, Pietro Pala, John Phoenix, Janice Piatt, Darren Russell, Eduard Sanders, Rachel Scott, Jae Sevelius, Hong Shang, Marc Siegel, Shobha Swaminathan, Vivian Tamayo, Darrell Tan, Allan Taylor, Bea Vuylsteke

**Affiliations:** Auckland District Health Board, Auckland, New Zealand; Duke University, Durham, NC, USA; University of California, San Francisco, San Francisco, CA, USA; David Geffen School of Medicine at UCLA, Los Angeles, CA, USA; Columbia University, New York, NY, USA; US Agency for International Development, Washington, DC, USA; Johns Hopkins University, Baltimore, MD, USA; Baylor College of Medicine, Houston, TX, USA; Universidad Nacional Mayor de San Marcos, Lima, Peru; University of Washington, Seattle, WA, USA; Universidade de São Paulo, São Paulo, Brazil; University of Alabama at Birmingham, Birmingham, AL, USA; University of Illinois, Chicago, IL, USA; University of British Columbia, Vancouver, BC, Canada; Jos University Teaching Hospital, Jos, Nigeria; University of Nairobi, Nairobi, Kenya; Hospital of the University of Pennsylvania, Philadelphia, PA, USA; Yale University, New Haven, CT, USA; The Chinese University of Hong Kong, Hong Kong, China; Beth Israel Deaconess Hospital, Boston, MA, USA; Institut de Recherche en Santé, de Surveillance Epidemiologique et de Formations, Dakar, Senegal; Yale University, New Haven, CT, USA; Men’s Health Foundation, Los Angeles, CA, USA; Infectious Diseases Institute, Makerere University, Kampala, Uganda; Medical Research Council/Uganda Virus Research Institute and London School of Hygiene & Tropical Medicine Uganda Research Unit, Entebbe, Uganda; Huntridge Family Clinic, Las Vegas, NV, USA; Phoenix Children’s Hospital, Phoenix, AZ, USA; Cairns and Hinterland Hospital and Health Service, Cairns, Australia; KEMRI Wellcome Trust, Kilifi, Kenya; Georgetown University, Washington, DC, USA; University of California San Francisco, San Francisco, CA, USA; The First Affiliated Hospital of China Medical University, Shenyang, China; George Washington University, Washington, DC, USA; New Jersey Medical School, Newark, NJ, USA; FDI Clinical Research, San Juan, Puerto Rico; University of Toronto, Toronto, ON, Canada; US Centers for Disease Control and Prevention, Atlanta, GA, USA; Institute of Tropical Medicine, Antwerp, Belgium; 1 UCLA Center for Clinical AIDS Research and Education, Los Angeles, California, USA; 2 Gilead Sciences, Inc, Foster City, California, USA; 3 Bridge HIV, San Francisco Department of Public Health, San Francisco, California, USA; 4 US Centers for Disease Control and Prevention, Atlanta, Georgia, USA; 5 University of Washington, Seattle, Washington, USA; 6 Instituto Nacional de Infectologia Evandro Chagas/Fundação Oswaldo Cruz (Fiocruz), Rio de Janeiro, Brazil; 7 UC San Diego Health, San Diego, California, USA; 8 State University of New York, New Paltz, New York, USA; 9 Fenway Health and Harvard Medical School, Boston, Massachusetts, USA; 10 Hunter College, New York, New York, USA; 11 The Desmond Tutu HIV Centre, University of Cape Town, Cape Town, South Africa; 12 Centre de Recherche du CHU de Québec– Université Laval, Quebec, Canada; 13 Public Health Service of Amsterdam, Amsterdam, Netherlands; 14 Department of Health Policy, Vanderbilt University School of Medicine, Nashville, Tennessee, USA; 15 MRC Clinical Trials Unit at University College London, London, United Kingdom; 16 Brown University, Providence, Rhode Island, USA; 17 Medical University of South Carolina, Charleston, North Carolina, USA; 18 The University of Alabama at Birmingham, Birmingham, Alabama, USA; 19 Department of Medicine, University of California San Francisco, San Francisco, California, USA

**Keywords:** pre-exposure prophylaxis, HIV-1, emtricitabine, tenofovir disoproxil fumarate

## Abstract

**Background:**

Oral pre-exposure prophylaxis (PrEP) with emtricitabine/tenofovir disoproxil fumarate (F/TDF) has high efficacy against HIV-1 acquisition. Seventy-two prospective studies of daily oral F/TDF PrEP were conducted to evaluate HIV-1 incidence, drug resistance, adherence, and bone and renal safety in diverse settings.

**Methods:**

HIV-1 incidence was calculated from incident HIV-1 diagnoses after PrEP initiation and within 60 days of discontinuation. Tenofovir concentrations in dried blood spots (DBS), drug resistance, and bone/renal safety indicators were evaluated in a subset of studies.

**Results:**

Among 17 274 participants, there were 101 cases with new HIV-1 diagnosis (.77 per 100 person-years; 95% confidence interval [CI]: .63–.94). In 78 cases with resistance data, 18 (23%) had M184I or V, 1 (1.3%) had K65R, and 3 (3.8%) had both mutations. In 54 cases with tenofovir concentration data from DBS, 45 (83.3%), 2 (3.7%), 6 (11.1%), and 1 (1.9%) had average adherence of <2, 2−3, 4−6, and ≥7 doses/wk, respectively, and the corresponding incidence was 3.9 (95% CI: 2.9–5.3), .24 (.060–.95), .27 (.12–.60), and .054 (.008–.38) per 100 person-years. Adherence was low in younger participants, Hispanic/Latinx and Black participants, cisgender women, and transgender women. Bone and renal adverse event incidence rates were 0.69 and 11.8 per 100 person-years, respectively, consistent with previous reports.

**Conclusions:**

Leveraging the largest pooled analysis of global PrEP studies to date, we demonstrate that F/TDF is safe and highly effective, even with less than daily dosing, in diverse clinical settings, geographies, populations, and routes of HIV-1 exposure.

Daily oral pre-exposure prophylaxis (PrEP) with emtricitabine/ tenofovir disoproxil fumarate (F/TDF) is highly effective against type 1 human immunodeficiency virus (HIV-1) acquisition in diverse populations, including those with different exposure routes [1, 2]. F/TDF efficacy is strongly linked to adherence; individuals with high adherence demonstrate high levels of protection against HIV-1 acquisition [3–7]. F/TDF adherence can be assessed objectively by measuring intracellular tenofovir diphosphate (TFV-DP) concentrations (the active form of the pro-drug tenofovir disoproxil fumarate [TDF]), in dried blood spots (DBS) prepared from individuals taking F/TDF [8, 9]. TFV-DP has a 17-day half-life in DBS, allowing assessment of F/TDF cumulative dosing for PrEP in the preceding 12 weeks [10–12]. Previous studies demonstrated that the TFV-DP concentration in DBSs remains above the prevention efficacy threshold in peripheral blood mononuclear cells for 2 weeks after F/TDF PrEP discontinuation [13, 14]. The relationship between TFV-DP concentration in DBS (reported as fmol/punch) and dosing frequency has been described previously [3, 11].

Following approval of F/TDF for PrEP in the United States [15–17], a series of prospective demonstration projects and clinical trials enrolling people using F/TDF for PrEP were conducted. These studies allowed generation of data regarding on-PrEP HIV-1 acquisition, drug resistance, adherence, risk factors for nonadherence, and bone and renal safety in global, diverse populations including those disproportionally affected by HIV-1, younger persons, transgender persons, cisgender women, and Black or African American and Hispanic or Latinx men who have sex with men (MSM).

Here we report an analysis of >17 000 individuals in 72 F/TDF PrEP studies across 28 countries from 2011 to 2020, evaluating HIV-1 incidence, drug resistance, PrEP adherence, and bone and renal safety.

## Methods

### Study Design and Population

Included studies are summarized in Supplementary Table 1. Each study’s procedures for enrollment of HIV-1-negative individuals included informed consent and approval by appropriate institutional review boards or similar authorities prior to implementation. Although funded via different mechanisms, all participants received F/TDF donated by Gilead Sciences, Inc (Foster City, CA). PrEP discontinuation reasons, including HIV-1 infection, adverse events (AEs), participant preferences, and missed visits or loss to follow-up, were defined in each study. In our analysis, the follow-up period was defined as the date of PrEP initiation until the earliest date of study completion, HIV-1 diagnosis, study-defined discontinuation, loss to follow-up, administrative censoring, or end of follow-up at 96 weeks. HIV-1 infections diagnosed within 60 days (~4 times the half-life of intracellular TFV-DP) of PrEP discontinuation were included. Temporary PrEP discontinuations were accounted for by recording dates for PrEP initiation, PrEP stop, and PrEP re-initiation, if any. The gap between each stop and re-initiation was not considered as on-PrEP time. We collected individual-level demographic and clinical information and HIV-1 infection status from each study. Some studies provided measurements of TFV-DP concentration in DBSs, resistance testing results for participants diagnosed with HIV-1, occurrence of renal AEs or bone fractures, or creatinine clearance. All available data were included in the analysis. We defined HIV-1 incidence rate (IR) as the number of incident HIV-1 diagnoses per 100 person-years of follow-up.

### Adherence Measurement

The TFV-DP concentrations in DBS were quantified by liquid chromatography/tandem mass spectrometry [8]. We developed a weighted-average drug concentration calculation that prioritizes TFV-DP concentration measurements from specimens collected near to the date of HIV-1 diagnosis. We decreased measurement *i*’s weight (*w_i_*) by 50% for every fraction of 4-week increments prior to detection of infection (ie, $w_{i}=50{\%}^{\left(\frac{t_{i}}{4\;weeks}\right)}$), resulting in the following formula for average TFV-DP concentration:

$$Average\left[TFV-DP\right]=\frac{\sum_{i=1}^{n}\left(x_{i}*50{\%}^{\left(\frac{t_{i}}{4\;weeks}\right)}\right)}{\sum_{i=1}^{n}50{\%}^{\left(\frac{t_{i}}{4\;weeks}\right)}}$$

where ***t***_1_ … ***t***_***n***_ represents the time in weeks from measurement 1 to the HIV-1 diagnosis date for the individual’s *n*th available measurement (*x*_*i*_). For those who did not acquire HIV-1 infection, ***t***_1_ … ***t***_***n***_ = 0, and the formula reduces to the simple arithmetic mean, reflecting equal importance of all measurements. We used weighted-average TFV-DP concentration to estimate the dosing frequency equivalent in number of doses per week: low adherence, <2 doses/week (<350 fmol/punch of TFV-DP); moderate adherence, 2–3 doses/week (350 to <700 fmol/punch); and high adherence, 4–6 doses/week (700 to <1250 fmol/punch) or 7 or more doses/week (≥1250 fmol/punch) [3, 11]. We excluded 1116 measurements from 518 individuals due to missing or pre-PrEP initiation sample-collection dates and 52 measurements from 29 individuals that were outside the assay’s reportable range.

### Adherence and Adherence Risk Factor Analyses

We analyzed population-level participant characteristics according to adherence level using mixed logistic regression with a random effect by study to describe associations between adherence and different participant characteristics. We used multilevel mixed modeling to examine weekly repeated measures of TFV-DP concentrations in DBS and to assess least-squares means of TFV-DP concentrations by HIV-1 infection status. This model was adjusted for age, gender, race/ethnicity, number of visits, HIV-1 infection status and its interaction with visit date, and random effect with the person nested in each individual protocol/study.

### Bone and Renal Safety Analyses

We calculated the incidence of nontraumatic bone fractures and adjudicated renal AEs per 100 person-years using the number of investigator-reported events over time from PrEP initiation for the incidence calculation. Renal and bone AEs were adjudicated and reviewed by the study team, excluding events with an etiology not biologically plausibly related to F/TDF use (eg, urinary tract pathology, renal calculus, bone fractures after trauma) (Supplementary Table 2). For individuals with laboratory creatinine data, we performed multilevel linear regression modeling (accounting for random effects of individuals in each study) to test associations between per a 100-fmol/punch increase in TFV-DP concentration in DBSs and per-unit increase in creatinine, per-unit decrease in creatinine clearance, and percentage change of creatinine from baseline at the same follow-up visit.

Four of the 72 studies included in our analysis offered participants an option for nondaily PrEP regimens [18–22]. We conducted sensitivity analyses excluding participants from these studies (n = 1801) to evaluate any differences in outcomes.

## Results

### Study Population

**Figure 1:**
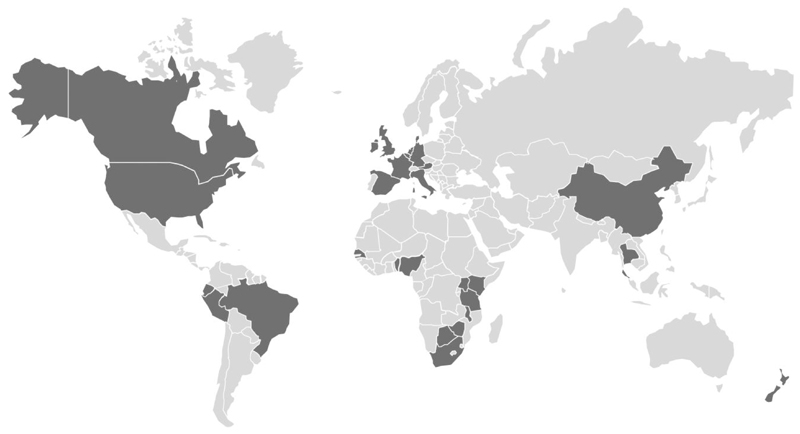
Map showing the 28 countries in which one or more F/TDF PrEP demonstration studies were conducted (dark gray shading): Austria, Belgium, Benin, Botswana, Brazil, Canada, China, Denmark, Germany, Ecuador, France, Ireland, Italy, Kenya, Malawi, Netherlands, New Zealand, Nigeria, Peru, Senegal, South Africa, Spain, Tanzania, Thailand, Uganda, United Kingdom, United States, and Zimbabwe. Abbreviations: F/TDF, emtricitabine/tenofovir disoproxil fumarate; PrEP, pre-exposure prophylaxis.

We collected data from 20 872 participants starting F/TDF for PrEP between June 2011 and September 2019 as part of 72 studies in 28 countries (Figure 1, Supplementary Table 1). We excluded participants missing data required for the calculation of on-PrEP follow-up time (n = 3574) and who were diagnosed with HIV-1 infection >60 days (corresponding to ~4 times the half-life of intracellular TFV-DP) after PrEP discontinuation (n = 24) from the analysis. The final analysis included 17 274 participants, with a median follow-up of 0.76 years (interquartile range [IQR], 0.26–1.40) and median age at PrEP initiation of 28 years (range, 14–77; IQR, 22–36). There were 10 946 cis-gender men (63%; median age, 30 years; IQR, 25–38), 5502 cis-gender women (32%; median age, 23 years; IQR, 20–30), 405 transgender women (2.3%; median age, 29 years; IQR, 24–36), and 337 transgender or nonbinary individuals for whom specific gender identity data were not available (2.0%; median age, 28 years; IQR, 23–36). Of the 17 274 participants, 4046 (23%) were non-Hispanic or Latinx White, 5626 (33%) were Black or African American, 1996 (12%) were Hispanic or Latinx, and 629 (43.6%) were Asian. Most participants lived in North America (6276, 36%) or Africa (5602, 32%), followed by South America (2017; 12%), Asia (1866; 11%), Europe (1360; 7.9%), and Oceania (153; 0.9%).

### HIV-1 Incidence Rates

In this dataset of 17 274 individuals, 101 acquired HIV-1 after PrEP initiation and within 60 days of PrEP discontinuation, with a mean follow-up of 0.59 years, resulting in an HIV-1 IR of .77 per 100 person-years (95% CI: .63–.94) (Table 1). The IR was 2.0 (95% CI: .73–5.2) for individuals aged 14–<18 years, 1.1 (95% CI: .88–1.3) for individuals aged 18–<35 years, and .20 (95% CI: .10–.39) for individuals aged ≥35 years (Table 1). Eighty-six (85%) participants discontinued PrEP on or after the date of HIV-1 diagnosis, 11 (11%) between 1 and 30 days before diagnosis, and 4 (4.0%) between 31 and 60 days before diagnosis. Among the 6598 individuals with available adherence data, 54 were diagnosed with HIV-1 infection; 45 (83.3%), 2 (3.7%), 6 (11.1%), and 1 (1.9%) of these individuals had an average adherence of <2, 2−3, 4−6, and ≥7 doses/week, respectively; the corresponding IRs were 3.9 (95% CI: 2.9–5.3), .24 (95% CI: .06–.96), .27 (95% CI: .12–.60), and .054 (95% CI: .008–.38) per 100 person-years. The IR was .73 per 100 person-years (95% CI: .57–.93) for cisgender men (n = 10 946), compared with .96 (95% CI: .68–1.4) for cisgender women (n = 5502) and .66 (95% CI: .16–2.6) for transgender women (n = 405).

### Drug Resistance

Results from resistance analyses on specimens collected at the time of diagnosis were available for 78 (77%) of the 101 HIV-1 cases. Among the 78 cases, 13 (17%) were identified within 1 month of F/TDF initiation, suggesting possible unrecognized baseline infection, and 7 (9.0%) were diagnosed >2 weeks after F/TDF discontinuation (Table 2). Of the 78 participants, 18 (23%) had M184IV (primary emtricitabine resistance mutation), 1 participant (1.3%) had K65R (primary tenofovir resistance mutation), and 3 participants (3.8%) had both (Table 2 and Supplementary Table 3). Twelve (15%) participants had additional non-polymorphic nucleoside or non-nucleoside reverse transcriptase inhibitor (NRTI or NNRTI) resistance-associated mutations (RAMs) (K103N, Y181C, or G190A; 5 also had M184V/I and/or K65R) (Supplementary Table 3). In virus samples from 47 (60.3%) of the 78 participants with resistance data, no non-polymorphic RAMs were detected. The TFV-DP concentration data in DBSs were available for 9 participants with M184V/I and/or K65R mutations.

Of these, 5 had low adherence (<2 doses/wk) and 4 had high adherence (≥4 doses/wk) based on TFV-DP concentration in DBS. The 4 high-adherence cases were diagnosed 28 to 84 days after PrEP initiation and were judged to be unrecognized baseline HIV-1 infections (Supplementary Table 3) [13, 14, 22].

### Adherence

One or more measurements of TFV-DP concentration in DBS were available from 6598 study participants. We classified 21%, 14%, 36%, and 29% of these participants as having received <2, 2−3, 4−6, or ≥7 doses of F/TDF per week (weighted-average drug concentration calculation), respectively (Table 3).

**Figure 2:**
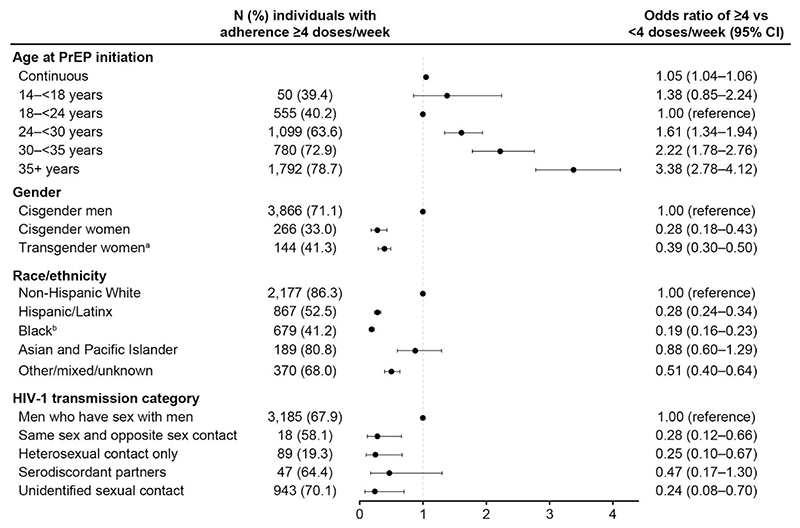
Odds ratios and 95% CIs for adherence of ≥4 versus <4 F/TDF doses per week (≥700 vs <700 fmol of tenofovir-diphosphate per punch in DBSs) in the subset of 6598 participants with DBS specimens available. ^a^Individuals were categorized as transgender, but no specific gender identity data were available. ^b^Includes individuals in the United States who identified as non-Hispanic or Latinx Black or African American. Abbreviations: CI, confidence interval; DBS, dried blood spot; F/TDF, emtricitabine/tenofovir disoproxil fumarate; HIV-1, type 1 human immunodeficiency virus; PrEP, pre-exposure prophylaxis.

Figure 2 shows the association between demographic or clinical characteristics with the likelihood of high adherence (≥4 doses/wk) among the 6598 individuals with available adherence data. Participants aged ≥30 years were approximately 2–3-fold more likely to have high adherence compared with those aged 18−24 years. Hispanic/Latinx and Black or African American individuals were approximately 3–5-fold less likely to have high adherence compared with White PrEP users. Individuals who had heterosexual contact were approximately 4-fold less likely to have high adherence compared with MSM.

**Figure 3:**
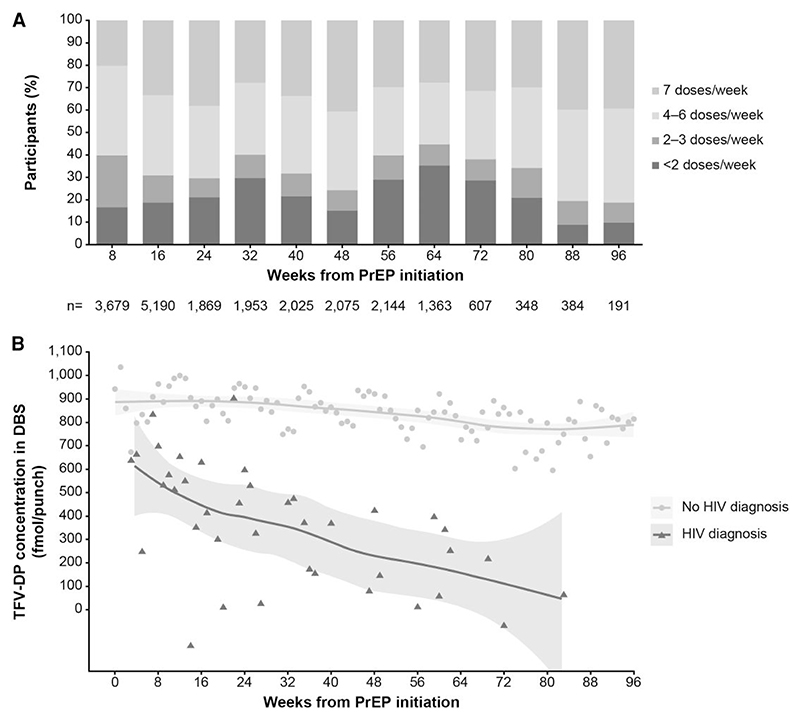
Adherence measured by TFV-DP in DBS. (*A*) Proportions of participants in each stratum of adherence as measured by intraerythrocytic TFV-DP in DBS samples. (*B*) Least-squares means by week for individuals diagnosed with HIV-1 and individuals not diagnosed. The trend of TFV-DP concentrations over time was depicted by fitting the data with a locally weighted scatterplot smoothing model. Abbreviations: DBS, dried blood spots; HIV, human immunodeficiency virus; PrEP, pre-exposure prophylaxis; TFV-DP, tenofovir diphosphate.

The proportion of participants in the 2 highest categories of adherence varied between 55%–80% over time (Figure 3A). Mean TFV-DP concentration (least-squares-means estimates calculated from the multilevel mixed model) decreased quickly after PrEP initiation among individuals who acquired HIV-1 during follow-up, compared with participants who did not (Figure 3B).

### Bone and Renal Safety

In 4969 individuals with available bone safety data, investigators reported nontraumatic bone fractures in 30 (0.60%) participants (Table 4). Mean age at nontraumatic bone fracture was 33 years (median, 29; IQR, 24–37). The IR for nontraumatic bone fractures was .69 (95% CI: .48–.98) per 100 person-years. In 5172 participants with renal safety data, 510 (9%) experienced adjudicated renal AEs at a mean age of 33 years (median, 29; IQR, 22–41). Among these, 247 (48.4%) were Grade 1/mild, 250 (49%) were Grade 2/moderate, and 13 (2.5%) were Grade 3/severe. The IR for adjudicated renal AEs was 11.8 (95% CI: 10.8–12.9) per 100 person-years. Of the 422 participants with additional details on renal AEs, 73 (17.3%) were likely related to the study drug (documented as “probably,” “possibly,” or “yes”).

**Figure 4:**
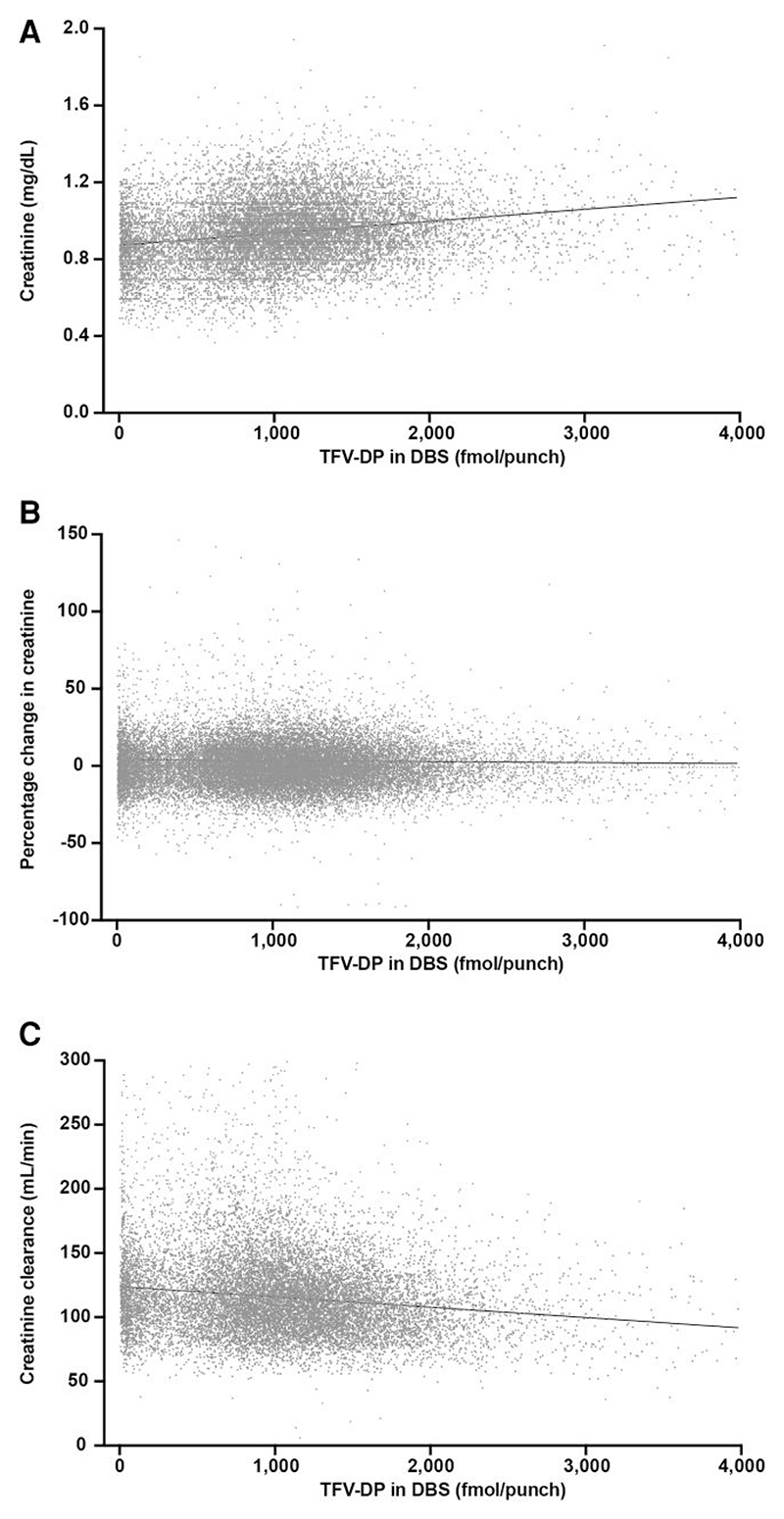
Creatinine and creatinine clearance versus TFV-DP in DBS measured in specimens collected on the same date. TFV-DP and creatinine concentration data at both baseline and at least 1 follow-up visit were available from 4595 individuals and 13 511 follow-up visits, and TFV-DP concentration and creatinine clearance data from 4417 individuals and 12 525 follow-up visits. (*A*) Creatinine concentration. (*B*) Percentage change in creatinine concentration versus baseline. (*C*) Creatinine clearance rate. Abbreviations: DBS, dried blood spots; TFV-DP, tenofovir diphosphate.

Associations between TFV-DP concentration in DBS and creatinine concentration, percentage change in creatinine concentration from baseline, and creatinine clearance in mL/min are shown in Figure 4. Each increase of 100 fmol/punch of TFV-DP in DBS was associated with a 0.005-mg/dL increase in creatinine concentration, a 0.15% increase in creatinine from baseline, and a 0.73-mL/min decrease in creatinine clearance from baseline.

### Sensitivity Analyses

Sensitivity analyses, comprising 15 473 participants, showed an overall HIV-1 IR of .78 (95% CI: .64–.95). Supplementary Table 4 summarizes HIV-1 IR for subgroups, revealing minimal differences from the main analysis. Excluding participants from the 4 trials with nondaily PrEP options did not change findings for the association between DBS-measured adherence and changes in creatinine and creatinine clearance, and IR for bone and adjudicated renal AEs.

## Discussion

Following Food and Drug Administration approval in 2012 and the establishment of safety and efficacy of daily oral F/TDF for the prevention of sexual transmission of HIV-1 in clinical trials, many studies were conducted to evaluate HIV-1 incidence, resistance, safety, and adherence in real-world settings. Over 17 000 individuals from 28 countries and 72 studies were included in the present analysis, which aggregated the experience of oral F/TDF use in racially, ethnically, and geographically diverse individuals. This is the largest such analysis of F/TDF PrEP and HIV-1 incidence and safety outcomes.

We confirmed the dose-dependent relationship between adherence and protective efficacy of F/TDF for PrEP. HIV-1 IR among participants with low adherence was high (3.9 per 100 person-years), indicating that the studies enrolled participants with a high likelihood of HIV-1 acquisition. Comparatively, HIV-1 IR was very low (.05 per 100 person-years) in participants with consistent daily dosing, corroborating the high PrEP efficacy observed in clinical trials. HIV-1 IRs among participants averaging 2–3 and 4–6 doses per week were intermediate but considerably lower than that observed in the low-adherence subgroup (.24 and .27 per 100 person-years, respectively). This highlights the potential benefit of PrEP in individuals who do not maintain daily dosing and corroborates the post hoc analyses from the Preexposure Prophylaxis Initiative (iPrEX) study that found that individuals who took ≥4 doses per week achieved a high level of protection against HIV-1 [3, 10].

Adherence was consistently better in older individuals, indicating that adolescents and young adults may require additional support to optimize PrEP use. Adherence was also lower among persons of color, cisgender women, transgender women, and heterosexual individuals. This highlights the need for improved efforts to address social, structural, and system-level access barriers to healthcare broadly, and specifically within HIV-1 prevention services. Prior studies have also identified individual-level challenges, such as individuals not perceiving a benefit from PrEP, or having other competing priorities that take precedence over sexual health and HIV-1 prevention [24–27]. While cisgender women and girls comprise a large proportion of new HIV-1 diagnoses globally [28], identifying and establishing objective assessments of who can benefit from PrEP among cisgender women outside of endemic settings remains a critical unmet research gap. More diverse PrEP modalities, including injectables, vaginal rings, and other agents, are needed to provide individuals with options that best address their needs.

A longstanding concern regarding the use of similar drugs for prevention and first-line HIV-1 treatment is that resistance selected by PrEP could decrease the efficacy of treatment should an individual on PrEP acquire HIV-1. In our analysis, mutations associated with resistance to emtricitabine and/or TDF were found in 28% of suspected incident cases. It is difficult to distinguish whether these mutations were present in the infecting virus or selected by F/TDF during PrEP. Mutations associated with resistance to other drugs in viruses from some participants suggests that, in certain cases, the infecting virus likely originated from a person with multidrug-resistant HIV-1, possibly resulting from combination antiretroviral therapy (ART) failure. Regardless of the origin of the resistance, results from clinical trials and observational cohorts indicate that replication of viruses bearing M184IV and/or K65R can be successfully suppressed with ART regimens, including dolutegravir or bictegravir with emtricitabine or lamivudine and 1 other NRTI [29–33]. The ability to use standard first-line ART regimens successfully following rare cases of PrEP failure is a critical consideration.

Our findings demonstrate a relationship between F/TDF adherence and small decreases in creatinine clearance, which, while unadjusted, are consistent with previous data. The rate of bone and renal AEs observed in this pooled analysis of approximately 5000 participants from multiple studies is consistent with the well-characterized bone and renal safety profile of F/TDF [34, 35]. However, this dataset was not designed to enable calculation of dose-dependent renal and bone-related AE rates, since bone and renal AEs were reported per individual study protocol, which may have been heterogeneous. Insufficient AE data limited our ability to fully characterize renal safety events; however, of those with available data, most renal AEs were Grade 2 or lower and unrelated to the study drug. This aligns with a recent meta-analysis that indicated that, while F/TDF use was associated with greater odds of experiencing renal AEs, these events were often mild and uncommon [34].

This study has several limitations. Pooling individual study results was not prospectively planned; each contributing study or project had a unique protocol, eligibility criteria with respect to sexual behavior, monitored safety parameters, and duration of follow-up. Specimens for adherence measurements were not always collected at the visit closest to the detection of HIV-1 infection, and resistance testing was not always available. Our incidence definition, using a 60-day window following PrEP discontinuation and a weighting function to emphasize TFV-DP concentration measurements collected near to the date of HIV-1 diagnosis, could have biased our results. Some studies included pregnant and postpartum women, for whom the correlation between TFV-DP concentration in DBSs and adherence may be different. Four of the 72 studies in our analysis included nondaily PrEP regimens. We were unable to identify specific regimens for these participants, limiting our ability to characterize outcomes separately for this small subgroup. Excluding these 4 studies in sensitivity analyses revealed no meaningful differences in outcomes. Further, limited participant follow-up restricts our ability to comment on predictors or correlates of longer-term adherence, persistence, and retention. Despite these limitations, this is the largest and most diverse pooled analysis of individuals using F/TDF for PrEP, supporting real-world use.

Evaluation of new PrEP strategies across a range of geographies, contexts, race/ethnicities, and in nonrandomized clinical trial settings is important to inform optimal implementation (including adherence support) strategies, and to characterize their efficacy and safety in less-controlled, real-world scenarios. These studies demonstrate that F/TDF for PrEP has a favorable safety profile, is highly effective when used as prescribed, and maintains some efficacy even with imperfect adherence.

## Supplementary Material

Supplementary materials are available at *Clinical Infectious* Diseases online. Consisting of data provided by the authors to benefit the reader, the posted materials are not copyedited and are the sole responsibility of the authors, so questions or comments should be addressed to the corresponding author.

Supplementary material

## Figures and Tables

**Table 1:** HIV-1 Incidence Rates After Up to 96 Weeks in 72 Post-Approval Studies of F/TDF for PrEP in 28 Countries, 2011–2021

	HIV-1 Diagnosis			No HIV-1 Diagnosis		
	n	Mean Person-years		n	Mean Person-years	IR (95% CI) per 100 Person-years
All (n = 17 274)	101	0.59		17 173	0.76	.77 (.63, .94)
Age at PrEP initiation 14 to <18 y (n = 368)	4	0.51		364	0.56	2.0 (.73, 5.2)
Age at PrEP initiation 18 to <35 y (n = 11 453)	87	0.58		11 366	0.70	1.1 (.88, 1.3)
Age at PrEP initiation 35+ y (n = 4766)	8	0.88		4758	0.85	.20 (.10, .39)
TFV-DP in DBSs not collected	47	0.40		10 629	0.66	.67 (.50, .89)
TFV-DP (fmol/punch) in DBSs available	54	0.76		6544	0.92	.89 (.68, 1.2)
<350 (<2 doses/wk)	45	0.81		1335	0.83	3.9 (2.9, 5.3)
350 to <700 (2–3 doses/wk)	2	0.9		934	0.89	.24 (.060, .96)
700 to <1250 (4–6 doses/wk)	6	0.35		2352	0.94	.27 (.12, .60)
≥1250 (≥7 doses/wk)	1^a^	0.67		1923	0.96	.054 (.008, .38)
Cisgender men (n = 10 946)	65	0.66		10 881	0.82	.73 (.57, .93)
Cisgender women (n = 5502)	33	0.41		5469	0.63	.96 (.68, 1.4)
Transgender women (n = 405)	2	1.03		403	0.75	.66 (.16, 2.6)
Transgender (n = 337)^b^	1	1.3		336	1.03	.29 (.041, 2.0)

Abbreviations: CI, confidence interval; DBS, dried blood spots; F/TDF, emtricitabine/tenofovir disoproxil fumarate; HIV-1, type 1 human immunodeficiency virus; IR, incidence rate; PrEP, pre-exposure prophylaxis; TFV-DP, tenofovir diphosphate.

a The participant, a cisgender man who had sex with men, had average TFV-DP DBS concentrations of 2234 and 2258 fmol/punch at 6 months after the start of F/TDF for PrEP and at time of HIV-1 diagnosis, respectively [23]; to date, this is the only reported infection with wild-type HIV infection while taking oral daily PrEP with documented daily adherence.

b Individuals were categorized as transgender, but no specific gender identity data were available.

**Table 2:** Resistance Data in Individuals Who Were Diagnosed With HIV-1 Infection after Initiation of F/TDF for PrEP in 72 Post-Approval Studies in 28 Countries, 2011–2021

	n With Resistance Data	n (%) With K65R and/ or M184IV	n (%) With Other Mutations^a^	n (%) With No Mutations
All	78	22 (28)	17 (22)	47 (60)
Days between PrEP initiation and HIV-1 diagnosis				
0 to 30^b^	13	6 (46)	3 (23)	6 (36)
31 to 60	7	3 (43)	2 (29)	2 (29)
61 to 180	20	8 (40)	4 (20)	11 (55)
>180	38	5 (13)	8 (21)	28 (74)
Days between PrEP stop and HIV-1 diagnosis				
<14	71	21 (30)	16 (22)	41 (58)
≥14	7	1 (14)	1 (14)	6 (86)
TFV-DP concentration in DBS (fmol/punch)				
No DBS sample	33	13 (39)	7 (21)	15 (45)
<350 (<2 doses/wk)	38	5 (13)	8 (21)	29 (76)
350 to <700 (2– 3 doses/wk)	2	0 (0)	0 (0)	2 (100)
700 to <1250 (4–6 doses/wk)	4	4 (100)	2 (50)	0 (0)
≥1250 (≥7 doses/wk)	1	0 (0)	0 (0)	1^c^ (100)

Abbreviations: DBS, dried blood spots; F/TDF, emtricitabine/tenofovir disoproxil fumarate; HIV-1, type 1 human immunodeficiency virus; PrEP, pre-exposure prophylaxis; TFV-DP, tenofovir diphosphate.

a Other mutations include those associated with resistance to nucleoside reverse transcriptase inhibitors (other than K65R or M184IV; M41L, D67N, K70R, L210W, T215D/I/S/V, and K219E/Q were observed) or to nonnucleoside reverse transcriptase inhibitors (K103N, E138A, Y181C, and G190A were observed). Numbers may not add up to category totals since 6 samples had both K65R/M184IV and other mutations. See Supplementary Table 3 for details.

b Undetected infection at PrEP initiation.

c See footnote “a” in Table 1.

**Table 3:** Participant Demographics According to Adherence Level in 72 Post-Approval Studies of F/TDF for PrEP in 28 Countries, 2011–2021

		TFV-DP Concentration (fmol/punch)				
	All	Not Available	<350 (<2 Doses/wk)	350 to <700 (2–3 Doses/wk)	700 to <1250 (4–6 Doses/wk)	≥1250 (≥7 Doses/wk)
N (% of those with DBS)	17 274	10 676	1380 (21)	936 (14)	2358 (36)	1924 (29)
Age						
Median (IQR) age at PrEP initiation, y	28 (22–36)	26 (22–34)	25 (21–31)	27 (22–34)	31 (25–38)	34 (28–45)
14 to <18 y, n (%)	368	241	44 (35)	33 (26)	39 (31)	11 (8.7)
18 to <24 y, n (%)	4810	3431	542 (39)	282 (20)	378 (27)	177 (13)
24 to <30 y, n (%)	4196	2469	380 (22)	248 (14)	649 (38)	450 (26)
30 to <35 y, n (%)	2447	1377	150 (14)	140 (13)	447 (42)	333 (31)
Over 35 y, n (%)	4766	2489	255 (11)	230 (10)	840 (37)	952 (42)
Unknown/missing, n (%)	687	669	9 (50)	3 (17)	5 (28)	1 (5.6)
Median (IQR) person-years on PrEP	0.76 (0.26–1.04)	0.48 (0.20–1.00)	0.95 (0.50–1.14)	0.96 (0.74–1.13)	0.93 (0.87–1.11)	0.92 (0.88–1.12)
Gender, n (%)						
Cisgender men	10 946	5512	895 (16)	673 (12)	2067 (38)	1799 (33)
Cisgender women	5502	4696	352 (44)	188 (23)	190 (24)	76 (9.4)
Transgender women	405	90	122 (39)	68 (22)	91 (29)	34 (11)
Transgender, not otherwise specified^a^	337	303	9 (26)	6 (18)	8 (24)	11 (32)
Other/unknown	84	75	2 (22)	1 (11)	2 (22)	4 (44)
Race/ethnicity, n (%)						
Non-Hispanic White	4046	1523	133 (5.3)	213 (8.4)	1051 (42)	1126 (45)
Black or African American^b^	5626	3979	597 (36)	371 (23)	487 (30)	192 (12)
Hispanic or Latinx^c^	1996	346	545 (33)	238 (14)	500 (30)	367 (22)
Asian and Pacific Islander	629	395	28 (12)	17 (7.3)	81 (35)	108 (46)
Other/mixed/unknown	4977	4433	77 (14)	97 (18)	239 (44)	131 (24)

Abbreviations: DBS, dried blood spot; F/TDF, emtricitabine/tenofovir disoproxil fumarate; IQR, interquartile range; PrEP, pre-exposure prophylaxis; TFV-DP, tenofovir diphosphate.

a Individuals were categorized as transgender, but no specific gender identity data were available.

b Includes individuals in the United States who identified as non-Hispanic or Latinx Black or African American.

c Hispanic or Latinx persons can be of any race.

**Table 4:** Incidence Rates for Investigator-Reported Nontraumatic Bone Fractures and Adjudicated Renal Adverse Events Within 96 Weeks of Follow-up in 72 Post-Approval Studies of F/TDF for Pre-exposure Prophylaxis in 28 Countries, 2011–2021

	Adverse Events Reported^a^			No Adverse Events Reported		
	n	Mean Person-years		n	Mean Person-years	IR (95% CI) Per 100 Person-years
Nontraumatic bone fractures						
All	30	0.46		4939	0.88	.69 (.48, .98)
No DBS sample	1	…		467	…	…
≤350 fmol/punch (<2 doses/wk)	12	0.27		1155	0.85	1.2 (.70, 2.1)
<350 to 700 fmol/punch (2–3 doses/wk)	2	0.31		656	0.90	.34 (.09, 1.4)
<700 to 1250 fmol/punch (4–6 doses/wk)	7	0.61		1527	0.93	.49 (.23, 1.0)
≥1250 (≥7 doses/wk)	8	0.60		1134	0.96	.73 (.37, 1.5)
Adjudicated renal adverse events						
All	510	0.44		4662	0.88	12 (11, 13)
No DBS sample	13	…		474	…	…
≤350 fmol/punch (<2 doses/wk)	155	0.61		1067	0.83	16 (13, 19)
350 to <700 fmol/punch (2–3 doses/wk)	61	0.49		676	0.89	9.6 (7.5, 12)
700 to <1250 fmol/punch (4–6 doses/wk)	151	0.30		1426	0.94	11 (9.3, 13)
≥1250 (≥7 doses/wk)	130	0.39		1019	0.96	13 (11, 15)

Abbreviations: CI, confidence interval; DBS, dried blood spot; F/TDF, emtricitabine/tenofovir disoproxil fumarate; IR, incidence rate; PrEP, pre-exposure prophylaxis.

a Bone and renal adverse events judged to be unrelated to PrEP were excluded (see Supplementary Table 2 for details).

## Data Availability

Gilead Sciences shares anonymized individual patient data upon request or as required by law or regulation with qualified external researchers based on submitted curriculum vitae and reflecting non conflict of interest. The request proposal must also include a statistician. Approval of such requests is at Gilead Science’s discretion and is dependent on the nature of the request, the merit of the research proposed, the availability of the data, and the intended use of the data. Data requests should be sent to datarequest@gilead.com.
