# Supplementary material for: Type 1 Human Immunodeficiency Virus (HIV-1) Incidence, Adherence, and Drug Resistance in Individuals Taking Daily Emtricitabine/Tenofovir Disoproxil Fumarate for HIV-1 Pre-exposure Prophylaxis: Pooled Analysis From 72 Global Studies
Source: Clin Infect Dis. 2024 Mar 14;79(5):1197–207. doi: 10.1093/cid/ciae143 (PMC7616831; doi:10.1093/cid/ciae143)
Supplement: ciae143_Supplementary_Data [file ciae143_supplementary_data.docx]

**Supplementary Table S1.** **Summary of the 72 Post-Approval Studies Using F/TDF for Oral PrEP Included in this Analysis.**

| **Study number** | **Study alias** | **Study title** | **Participants in the study** | **Participants in this analysis** | **Participants with adherence measures in dried blood spots** | **Countries (N)** | **Gender** | **Age range** | **New HIV-1 infections** | **Individuals with AEs** | **Study start** | **Study end** | **clinicaltrials.gov and publication URL** |
| --- | --- | --- | --- | --- | --- | --- | --- | --- | --- | --- | --- | --- | --- |
| CO-AU-276-4099 | NZ PrEP | Provision of HIV pre-exposure prophylaxis to men who have sex with men at high risk of acquiring HIV in a sexual health clinic setting- a demonstration project | 103 | 103 | 0 | NZL (N=103) | M | 19–55 | 0 | NA | 20/02/2017 | 21/08/2017 | https://www.ncbi.nlm.nih.gov/pmc/articles/PMC6597636/ |
| CO-US-164-0403 | HPTN 067/ADAPT | The ADAPT study: a phase II, randomized, open-label, pharmacokinetic and behavioral study of the use of intermittent oral emtricitabine/tenofovir disoproxil fumarate pre-exposure prophylaxis (PrEP) | 178 | 176 | 56 | THA (N=60), USA (N=57), ZAF (N=59) | F, M, T | 18–58 | 0 | 2 FRX 24 RAE | 15/09/2011 | 03/01/2015 | https://clinicaltrials.gov/ct2/show/NCT01327651 https://academic.oup.com/cid/article/66/11/1712/4840078; <https://www.ncbi.nlm.nih.gov/pmc/articles/PMC6107917/> |
| CO-US-164-0404 | iPrEx OLE | Open label extension of chemoprophylaxis for HIV prevention in men | 1,225 | 1,200 | 1,200 | BRA (N=191), ECU (N=151), PER (N=544), THA (N=54), USA (N=220), ZAF (N=40) | M, T | 19–70 | 22 | 10 FRX 11 RAE | 13/06/2011 | 11/12/2013 | https://clinicaltrials.gov/ct2/show/NCT00458393  <https://www.ncbi.nlm.nih.gov/pmc/articles/PMC6107918/> |
| CO-US-164-0432 | DAIDS PrEP Demo | Implementation of HIV pre-exposure prophylaxis (PrEP): A demonstration project | 557 | 455 | 455 | USA (N=455) | M, T, O | 18–66 | 1 | 3 FRX 9 RAE | 01/10/2012 | 22/01/2015 | https://www.ncbi.nlm.nih.gov/pmc/articles/PMC3942317/ |
| CO-US-164-0441 | CDC PrEP Demo (SHIPP) | Sustainable health center implementation PrEP pilot study | 2,186 | 1,808 | 1,038 | USA (N=1808) | F, M, T, O | 18–77 | 6 | NA | 29/09/2014 | 04/04/2018 | https://clinicaltrials.gov/ct2/show/NCT02074891  https://link.springer.com/article/10.1007/s10461-021-03388-5 |
| CO-US-164-0450 | EPIC PrEP | A randomized trial of PrEPmate, a PrEP adherence intervention for young msm in the us: enhancing PrEP in communities (EPIC) | 121 | 115 | 115 | USA (N=115) | F, M, T, O | 18–30 | 0 | 2 FRX | 01/04/2015 | 19/12/2016 | https://clinicaltrials.gov/ct2/show/NCT02371525  <https://academic.oup.com/cid/article/68/12/2010/5098440> |
| CO-US-164-0451 | CDC Botswana PrEP OLE | Open label extension (OLE) of the study of the safety and efficacy of daily oral antiretroviral use for the prevention of HIV infection in heterosexually active young adults in Botswana | 229 | 229 | 0 | BWA (N=229) | F, M | 23–44 | 0 | NA | 04/03/2013 | 01/05/2015 | https://clinicaltrials.gov/ct2/show/NCT04318210  <https://www.cdc.gov/nchhstp/newsroom/docs/prep-heterosexuals-factsheet.pdf> |
| CO-US-164-0452 | ATN 110 | Project PrEPare - an open label demonstration project and phase II safety study of pre-exposure prophylaxis use among young men who have sex with men (YMSM) in the United States | 200 | 175 | 175 | USA (N=175) | M | 18–23 | 4 | 5 FRX | 10/01/2013 | 01/01/2015 | https://clinicaltrials.gov/ct2/show/NCT01772823  https://www.ncbi.nlm.nih.gov/pmc/articles/PMC5140725/ |
| CO-US-164-0454 | PROUD | Pre-exposure option for reducing HIV in the UK: an open-label randomisation to immediate or deferred daily Truvada for HIV negative gay men | 468 | 462 | 0 | GBR (N=462) | M | NA | 1 | NA | 20/12/2012 | 16/04/2016 | https://clinicaltrials.gov/ct2/show/NCT02065986  https://www.ncbi.nlm.nih.gov/pmc/articles/PMC4700047/ |
| CO-US-164-0455 | ATN 113 | Project PrEPare an open label demonstration project and phase II safety study of pre-exposure prophylaxis use among 15- to 17-year-old men who have sex with men (MSM) in the United States | 79 | 66 | 66 | USA (N=66) | M | 15–18 | 2 | NA | 01/08/2013 | 14/10/2015 | https://clinicaltrials.gov/ct2/show/NCT01769456  <https://www.ncbi.nlm.nih.gov/pmc/articles/PMC5710370/> |
| CO-US-164-0461 | CHAMPS/Pluspills | CHAMPS: Choices for adolescent methods of prevention in South Africa | 148 | 148 | 0 | ZAF (N=148) | F, M | 14–20 | 1 | NA | 28/04/2015 | 23/01/2017 | https://clinicaltrials.gov/ct2/show/NCT02213328  https://www.ncbi.nlm.nih.gov/pmc/articles/PMC9832157/ |
| CO-US-164-0468 | Partners PrEP Demo | An open-label, pilot demonstration and evaluation project of antiretroviral-based HIV-1 prevention among high-risk HIV-1 serodiscordant African couples | 1,013 | 326 | 0 | KEN (N=177), UGA (N=149) | F, M | 19–64 | 13 | 1 FRX | 27/11/2012 | 30/05/2016 | https://clinicaltrials.gov/ct2/show/NCT02775929  https://www.ncbi.nlm.nih.gov/pmc/articles/PMC4995047/ |
| CO-US-164-0471 | PrEPared & Strong | PrEP for Black MSM: Community-based ethnography and clinic-based treatment | 204 | 204 | 0 | USA (N=204) | M | 14–59 | 0 | NA | 27/04/2014 | 14/08/2017 | https://clinicaltrials.gov/ct2/show/NCT02167386  https://link.springer.com/article/10.1007/s10461-020-02901-6 |
| CO-US-164-0478 | ALERT | CCTG 595: Text messaging intervention to improve adherence to PrEP in high-risk MSM | 398 | 357 | 357 | USA (N=357) | M, T | 19–64 | 2 | NA | 25/02/2013 | 09/02/2016 | https://www.clinicaltrials.gov/ct2/show/NCT01761643  https://www.ncbi.nlm.nih.gov/pmc/articles/PMC6248545/ |
| CO-US-164-0480 | PATH - PrEP | A pilot demonstration project to operationalize pre-exposure prophylaxis as part of combination HIV prevention among men who have sex with men (MSM) and transgender women in Los Angeles County | 301 | 279 | 279 | USA (N=279) | M, T | 21–69 | 0 | 2 FRX 161 RAE | 24/06/2013 | 26/05/2016 | https://www.clinicaltrials.gov/ct2/show/NCT01781806  https://www.ncbi.nlm.nih.gov/pmc/articles/PMC5681370/ |
| CO-US-164-0481 | Project PrEPare | Optimizing antiretroviral-based prevention by enhancing PrEP adherence in MSM | 50 | 50 | 0 | USA (N=50) | M | 20–68 | 0 | NA | 27/11/2012 | 06/06/2014 | https://clinicaltrials.gov/ct2/show/NCT01632397 |
| CO-US-164-0483 | HPTN 073 | Pre-exposure prophylaxis (PrEP) initiation and adherence among Black men who have sex with men (BMSM) in three United States cities | 226 | 167 | 167 | USA (N=167) | M | 19–69 | 2 | 1 FRX | 19/08/2013 | 22/09/2015 | https://clinicaltrials.gov/ct2/show/NCT01808352  https://www.ncbi.nlm.nih.gov/pmc/articles/PMC6792108/ |
| CO-US-164-1265 | SPARK | Intervention to enhance PrEP uptake and adherence in a community-based setting | 300 | 296 | 296 | USA (N=296) | M, T | 18–63 | 0 | NA | 15/01/2014 | 08/10/2016 | https://clinicaltrials.gov/ct2/show/NCT02037594  https://link.springer.com/article/10.1007/s10461-018-2376-y |
| CO-US-276-0108 | Brazilian PrEP | Implementation of the pre-exposure prophylaxis (PrEP) to HIV: a demonstrative project | 749 | 714 | 665 | BRA (N=714) | M, T | 18–67 | 3 | 2 FRX | 04/06/2014 | 04/02/2020 | https://clinicaltrials.gov/ct2/show/NCT01989611  https://www.sciencedirect.com/science/article/pii/S2352301818300080?via%3Dihub |
| CO-US-276-0115 | PrEP Uptake STD clinics | Implementation of HIV PrEP at STD clinics in Providence, Rhode Island and Jackson, Mississippi | 97 | 97 | 0 | USA (N=97) | F, M, T | 18–64 | 1 | NA | NA |  | https://www.ncbi.nlm.nih.gov/pmc/articles/PMC5129653/ |
| CO-US-276-0117 | SAFER | The impact and cost-effectiveness of safer conception strategies for HIV-discordant couples | 19 | 19 | 0 | ZWE (N=19) | F, M | 21–41 | 0 | NA | 27/03/2017 | 11/04/2019 | https://clinicaltrials.gov/ct2/show/NCT03049176  https://www.ncbi.nlm.nih.gov/pmc/articles/PMC7552466/ |
| CO-US-276-0127 | Trans PrEP | TransPrEP: social network-based PrEP adherence for transgender women in Peru | 89 | 89 | 0 | PER (N=89) | T | 19–58 | 0 | NA | 03/11/2017 | 02/08/2018 | https://clinicaltrials.gov/ct2/show/NCT02710032  https://www.ncbi.nlm.nih.gov/pmc/articles/PMC8084919/ |
| CO-US-276-1263 | Senegal FSW PrEP | A demonstration project of HIV pre-exposure prophylaxis (PrEP) with tenofovir DF/emtricitabine (TDF/FTC) among female sex workers in Dakar, Senegal | 273 | 271 | 0 | SEN (N=271) | F | 18–59 | 0 | NA | 16/07/2015 | 30/04/2016 | https://clinicaltrials.gov/ct2/show/NCT02474303  https://www.ncbi.nlm.nih.gov/pmc/articles/PMC7750667/ |
| CO-US-276-1264 | PMR_3Ps (Bekker/Celum) | A pilot prospective cohort evaluation of uptake and adherence to PrEP in young South African women | 200 | 200 | 181 | ZAF (N=200) | F | 16–25 | 1 | NA | 23/05/2017 | 06/04/2019 | https://clinicaltrials.gov/ct2/show/NCT03142256  https://www.ncbi.nlm.nih.gov/pmc/articles/PMC7196718/ |
| CO-US-276-1317 | MP3 Kenya | Gender-specific combination HIV prevention for youth in high burden settings (MP3-Youth) | 28 | 28 | 0 | KEN (N=28) | F | 18–24 | 0 | NA | 24/03/2015 | 06/05/2016 | https://clinicaltrials.gov/ct2/show/NCT01571128  https://www.ncbi.nlm.nih.gov/pmc/articles/PMC5434770/ |
| CO-US-276-1318 | Benin PrEP/TasP | Demonstration project of early antiretroviral therapy and pre-exposure prophylaxis for HIV prevention among female sex workers in Cotonou, Benin | 256 | 219 | 0 | BEN (N=219) | F | 18–59 | 0 | NA | 02/10/2014 | 29/01/2017 | https://clinicaltrials.gov/ct2/show/NCT02237027  https://www.ncbi.nlm.nih.gov/pmc/articles/PMC7803451/ |
| CO-US-276-1338 | CRUSH Demo | Connecting resources for urban sexual health | 257 | 257 | 0 | USA (N=257) | F, M, T, O | 17–30 | 0 | NA | 18/02/2014 | 25/11/2016 | https://clinicaltrials.gov/ct2/show/NCT02183909  https://blogs.bmj.com/bmj/2019/02/26/unreported-clinical-trial-of-the-week-resources-for-urban-sexual-health-with-an-hiv-prophylaxis-component-nct02183909/ |
| CO-US-276-1340 | Women's PrEP Demo | Pre-exposure prophylaxis to prevent HIV acquisition in US women: a demonstration project | 174 | 173 | 0 | USA (N=173) | F | 19–62 | 0 | NA | 28/02/2017 | 26/02/2021 | https://clinicaltrials.gov/ct2/show/NCT03058835 |
| CO-US-276-1636 | South Africa TAPS Demo | Expanded use of ART for treatment and prevention for female sex workers in South Africa | 219 | 219 | 0 | ZAF (N=219) | F | 17–55 | 0 | NA | 31/03/2015 | 30/06/2017 | https://www.ncbi.nlm.nih.gov/pmc/articles/PMC5051507/ |
| CO-US-276-1637 | COPE4YMSM | Effectiveness and cost effectiveness of a multi-level combination HIV preventive intervention with and without daily oral Truvada pre-exposure prophylaxis (PrEP) with adherence support among young men who have sex with men (YMSM) aged 18-26 in Bangkok and Pattaya, Thailand | 451 | 451 | 0 | THA (N=451) | F, M, T, O | 17–27 | 0 | NA | 10/10/2017 | 17/06/2021 | https://www.ncbi.nlm.nih.gov/pmc/articles/PMC7011123/ |
| CO-US-276-1639 | HPTN 082/HERS | Uptake and adherence to daily oral PrEP as a primary prevention strategy for young African women: a Vanguard Study | 427 | 412 | 390 | ZAF (N=269), ZWE (N=143) | F | 16–25 | 4 | 1 FRX 187 RAE | 12/10/2016 | 25/10/2018 | https://clinicaltrials.gov/ct2/show/NCT02732730  https://www.ncbi.nlm.nih.gov/pmc/articles/PMC7060297/ |
| CO-US-276-1691 | Nigeria PrEP | A demonstration project of antiretroviral-based HIV-1 prevention among HIV-1 serodiscordant couples in Nigeria | 342 | 342 | 0 | NGA (N=342) | F, M, O | 18–70 | 0 | NA | 24/11/2015 | 13/08/2017 | https://journals.plos.org/plosone/article?id=10.1371/journal.pone.0268011 |
| CO-US-276-1694 | Brazil Prevention | Combined use of HIV prevention methods and prophylaxis before and after consensual sexual exposure in Brazil: protocol for a pragmatic clinical trial at public healthcare clinics | 296 | 296 | 0 | BRA (N=296) | F, M, T | 18–70 | 0 | NA | 07/11/2016 | 10/08/2017 | https://bmjopen.bmj.com/content/5/8/e009021.long |
| CO-US-276-1710 | PREPPIE | HIV pre-exposure prophylaxis priming of immune effectors | 166 | 166 | 0 | UGA (N=166) | F | 17–51 | 1 | NA | 02/06/2017 | 17/07/2019 | https://clinicaltrials.gov/ct2/show/NCT02593409  https://www.sciencedirect.com/science/article/pii/S2352827321000215 |
| CO-US-276-1712 | AMPrEP | Biomedical interventions for HIV prevention in MSM in Amsterdam: a demonstration project | 376 | 324 | 324 | NLD (N=324) | M, T | 19–73 | 2 | 27 RAE | 28/08/2015 | 29/03/2019 | https://www.clinicaltrialsregister.eu/ctr-search/search?query=2014-002569-32  https://journals.lww.com/aidsonline/Fulltext/2021/09010/Improving_adherence_to_daily_preexposure.12.aspx |
| CO-US-276-1719 | IMPAACT 2009 | Pharmacokinetics, feasibility, acceptability, and safety of oral pre-exposure prophylaxis for primary HIV prevention during pregnancy and postpartum in adolescents and young women and their infants | 40 | 40 | 0 | USA, ZAF, ZWE, MWI, UGA (N=40) | F | 16–24 | 0 | NA | NA |  | https://clinicaltrials.gov/ct2/show/NCT03386578  https://www.ncbi.nlm.nih.gov/pmc/articles/PMC8492211/ |
| CO-US-276-1733 | PMR_Tanzania PrEP | Dyadic-based diagnosis, care, & prevention for HIV discordant couples in Tanzania | 64 | 64 | 0 | TZA (N=64) | F, M | 22–72 | 1 | NA | 23/11/2017 | 06/09/2019 | https://clinicaltrials.gov/ct2/show/NCT03098693  https://bmcpublichealth.biomedcentral.com/articles/10.1186/s12889-021-10707-x |
| CO-US-276-1774 | PMR_POWER | A cohort for evaluation of open-label PrEP delivery among Kenyan and South African Women: The POWER cohort | 1,781 | 1,781 | 0 | KEN, ZAF (N=1781) | F | 16–25 | 12 | NA | 14/06/2017 | 18/08/2019 | https://clinicaltrials.gov/ct2/show/NCT03490058  https://www.ncbi.nlm.nih.gov/pmc/articles/PMC9278271/ |
| CO-US-276-1806 | PMR_MPYA | Next generation real-time monitoring to assess PrEP adherence for young women | 348 | 347 | 0 | KEN (N=347) | F | 18–24 | 2 | NA | 21/12/2016 | 08/08/2019 | https://clinicaltrials.gov/ct2/show/NCT02915367  https://www.ncbi.nlm.nih.gov/pmc/articles/PMC8289198/ |
| CO-US-276-1836 | PUMP | A non-randomized, unblinded, retrospective and prospective analysis of 340 patients prescribed PrEP at The Southern California Men’s Medical Group | 60 | 59 | 0 | USA (N=59) | M, T | 20–73 | 0 | NA | 15/08/2016 | 29/07/2020 | NA |
| CO-US-276-1862 | Belgium PrEP | Be-PrEP-ared: HIV prevention with pre-exposure prophylaxis – a demonstration project in high risk men having sex with men in Belgium | 200 | 200 | 0 | BEL (N=200) | M, T | 22–70 | 0 | NA | 06/10/2015 | 15/05/2018 | https://www.clinicaltrialsregister.eu/ctr-search/search?query=2015-000054-37  https://www.ncbi.nlm.nih.gov/pmc/articles/PMC5306610/ |
| CO-US-276-1976 | CRUSH Women | A demonstration project examining interest in and uptake of HIV pre-exposure prophylaxis with Truvada among women attending community health clinics in Oakland | 31 | 18 | 18 | USA (N=18) | F, T | 22–69 | 0 | NA | 13/05/2016 | 29/08/2017 | https://clinicaltrials.gov/ct2/show/NCT02852226  https://www.californiaaidsresearch.org/files/award-abstracts/prevention-and-linkage-to-care/crush-prep-women.html |
| CO-US-276-2003 | AEGIS | PrEP adherence enhancement guided by iTAB and drug levels for women (AEGIS) | 136 | 118 | 118 | USA (N=118) | F | 19–67 | 0 | 61 RAE | 09/06/2016 | 25/09/2019 | https://clinicaltrials.gov/ct2/show/NCT02584140  https://www.ncbi.nlm.nih.gov/pmc/articles/PMC8905235/ |
| CO-US-276-2004 | Peru PrEP | Demonstration  project on the feasibility to implement a pre-exposure oral prophylaxis program in men who have sex with other men and transgender women at risk of acquiring HIV | 32 | 32 | 0 | PER (N=32) | M | 21–49 | 0 | NA | 23/01/2017 | 06/08/2017 | https://clinicaltrials.gov/ct2/show/NCT03043326 |
| CO-US-276-2060 | SCIP | Pilot of an mHealth-enhanced, safer conception intervention to reduce HIV-1 risk among Kenyan HIV-1 serodiscordant couples | 74 | 74 | 0 | KEN (N=74) | F, M | 20–60 | 0 | NA | 23/03/2016 | 03/08/2017 | https://clinicaltrials.gov/ct2/show/NCT03030768  https://www.ncbi.nlm.nih.gov/pmc/articles/PMC6452026/ |
| CO-US-276-2061 | Safer Conception SA | Safer conception for women: PrEP uptake/adherence to reduce periconception HIV risk for South African women | 143 | 138 | 0 | ZAF (N=138) | F | 19–36 | 3 | NA | 17/12/2017 | 09/07/2021 | https://clinicaltrials.gov/ct2/show/NCT03194308  https://www.ncbi.nlm.nih.gov/pmc/articles/PMC6661571/ |
| CO-US-276-2107 | DOT Diary Pilot | DOT diary longitudinal pilot: a mobile app for pre-exposure prophylaxis adherence in young men | 100 | 100 | 0 | USA (N=100) | M, O | 18–35 | 0 | NA | 11/02/2019 | 18/08/2019 | https://clinicaltrials.gov/ct2/show/NCT03771638  https://www.ncbi.nlm.nih.gov/pmc/articles/PMC9908647/ |
| CO-US-276-3919 | EMPOWERing | Project EMPOWERING: evidence-based PrEP for justice-involved women and their risk networks | 25 | 25 | 0 | USA (N=25) | F, M | 30–53 | 0 | NA | 30/01/2018 | 18/08/2019 | https://clinicaltrials.gov/ct2/show/NCT03293290  https://link.springer.com/article/10.1007/s10461-022-03709-2 |
| CO-US-276-3947 | Safer Conception UG | Adherence to periconception HIV risk-reduction among uninfected women in rural Uganda | 105 | 105 | 0 | UGA (N=105) | F | 20–71 | 1 | NA | 13/12/2017 | 05/01/2021 | https://clinicaltrials.gov/ct2/show/NCT03832530  https://www.ncbi.nlm.nih.gov/pmc/articles/PMC8944216/ |
| CO-US-276-3958 | Tambua Mapema Plus | Impact of a novel screening program to detect acute and prevalent HIV infection and reduce HIV transmission | 3 | 3 | 0 | KEN (N=3) | M | 27–37 | 0 | NA | 20/09/2018 | 07/08/2019 | https://clinicaltrials.gov/ct2/show/NCT03508908  https://www.ncbi.nlm.nih.gov/pmc/articles/PMC7442943/ |
| CO-US-276-4012 | DuDHS | Use of tenofovir/emtricitabine with immediate or deferred doxycycline 100mg PO daily for combination HIV and syphilis pre-exposure prophylaxis in HIV-negative men who have sex with men: a pilot study of dual daily HIV and syphilis PrEP (The DuDHS Trial) | 52 | 52 | 0 | CAN (N=52) | M, T | 20–69 | 0 | NA | 13/06/2018 | 18/08/2019 | https://clinicaltrials.gov/ct2/show/NCT02844634  https://www.ncbi.nlm.nih.gov/pmc/articles/PMC6886914/ |
| CO-US-276-4045 | PMR_Stay | The Stay Study: a demonstration project advancing PrEP delivery in the San Francisco Bay area transgender community | 159 | 159 | 0 | USA (N=159) | F, M, T, O | 18–73 | 0 | NA | 08/08/2017 | 02/04/2021 | https://clinicaltrials.gov/ct2/show/NCT03120936  https://journals.lww.com/jaids/Fulltext/2021/12151/Expanding_the_Pie_Differentiated_PrEP_Delivery.6.aspx |
| CO-US-276-4047 | PMR_TRIUMPH | TRIUMPH: trans research-informed communities united in mobilization for the prevention of HIV | 167 | 167 | 0 | USA (N=167) | T, O | 18–58 | 0 | NA | 09/10/2017 | 18/08/2019 | https://journals.lww.com/jaids/Fulltext/2021/12151/Uptake,_Retention,_and_Adherence_to_Pre_exposure.5.aspx |
| CO-US-276-4263 | CFAR PrEP 2 | The effect of social media support and financial incentives on adherence to HIV pre-exposure prophylaxis in young MSM of color in Washington, DC | 53 | 53 | 0 | USA (N=53) | M | 18–29 | 0 | NA | 17/04/2017 | 26/03/2021 | https://clinicaltrials.gov/ct2/show/NCT03078153  https://www.ncbi.nlm.nih.gov/pmc/articles/PMC7777911/ |
| CO-US-276-4325 | CROPrEP | A prevention multi-center study for HIV PrEP in MSM | 1,169 | 1,153 | 0 | CHN (N=1153) | M | 20–64 | 3 | NA | 11/12/2018 | 02/10/2019 | https://www.ncbi.nlm.nih.gov/pmc/articles/PMC6694544/ |
| CO-US-276-4368 | PMR_HK Crossover | Pre-exposure prophylaxis with on-demand versus daily TDF/FTC in MSM at high risk of HIV infection - a crossover study | 148 | 148 | 0 | CHN (N=148) | M | 19–61 | 1 | NA | 21/10/2017 | 12/10/2019 | https://www2.ccrb.cuhk.edu.hk/registry/public/534  https://www.ncbi.nlm.nih.gov/pmc/articles/PMC8412015/ |
| CO-US-276-4422 | Baltimore BMSM | A comparative effectiveness demonstration project for linkage and retention in PrEP care for men who have sex with men (PCA) | 12 | 11 | 0 | USA (N=11) | M | 22–46 | 0 | NA | 05/05/2015 | 23/05/2021 | https://clinicaltrials.gov/ct2/show/NCT03442192 |
| CO-US-276-4476 | PEP to PrEP Vegas | What happens in Vegas: identifying opportunities to expand an HIV prevention network to improve PEP and PrEP delivery | 35 | 35 | 0 | USA (N=35) | F, M, T | 19–61 | 0 | NA | 04/10/2018 | 29/09/2019 | https://clinicaltrials.gov/ct2/show/NCT03775083 |
| CO-US-276-4528 | Empower Study | HIV self-testing to empower prevention choices in sex workers | 1 | 1 | 0 | UGA (N=1) | F | 37–37 | 1 | NA | 06/07/2018 | 15/04/2019 | https://clinicaltrials.gov/ct2/show/NCT03426670  https://www.ncbi.nlm.nih.gov/pmc/articles/PMC8412089/ |
| CO-US-276-4549 | PMR_Women PrEP Cascade | Identification of the pre-exposure prophylaxis (PrEP) cascade for women and integration of PrEP into women's family planning services: a prospective cohort | 11 | 11 | 0 | USA (N=11) | F | 19–45 | 0 | NA | 17/10/2017 | 18/01/2021 | https://clinicaltrials.gov/ct2/show/NCT03705663 |
| CO-US-276-4550 | Phoenix Youth PrEP | Evaluation of implementation of the Phoenix PrEP Access Project for youth aged 13–24 | 23 | 23 | 0 | USA (N=23) | M, T | 15–24 | 0 | NA | 29/06/2015 | 17/08/2019 | https://clinicaltrials.gov/ct2/show/NCT03637322 |
| CO-US-276-4556 | PMR_Uganda PMTCT B+ | HIV self-testing of men to increase testing and prevention uptake among male partners and improve postpartum ART use in PMTCT B+ programs in Uganda | 62 | 56 | 0 | UGA (N=56) | M | 20–56 | 0 | NA | 12/06/2018 | 17/08/2019 | https://clinicaltrials.gov/ct2/show/NCT03484533  https://www.ncbi.nlm.nih.gov/pmc/articles/PMC8249052/ |
| CO-US-276-4557 | Kampala Bone Study | Impact of concurrent initiation of DMPA contraception and tenofovir PrEP on bone loss in young women | 317 | 315 | 0 | UGA (N=315) | F | 16–25 | 0 | NA | 06/06/2018 | 18/08/2019 | https://clinicaltrials.gov/ct2/show/NCT03464266  https://www.tandfonline.com/doi/full/10.1080/09540121.2023.2177250 |
| GS-US-412-2055 | DISCOVER | A phase 3, randomized, double-blind study to evaluate the safety and efficacy of emtricitabine and tenofovir alafenamide (F/TAF) fixed-dose combination once daily for pre-exposure prophylaxis in men and transgender women who have sex with men and are at risk of HIV-1 infection | 2,693 | 675 | 675 | AUT (N=19), CAN (N=34), DEU (N=87), DNK (N=42), ESP (N=74), FRA (N=5), GBR (N=103), IRL (N=20), ITA (N=8), NLD (N=16), USA (N=267) | M, T | 18–71 | 11 | 1 FRX 30 RAE | 13/09/2016 | 11/12/2019 | https://clinicaltrials.gov/ct2/show/NCT02842086  https://www.ncbi.nlm.nih.gov/pmc/articles/PMC9665936/ |
| IN-AU-164-1888 | QPrEP | The Queensland pre-exposure prophylaxis demonstration project | 50 | 50 | 0 | AUT (N=50) | M | NA | 0 | NA | 09/09/2015 | 12/11/2016 | https://www.anzctr.org.au/Trial/Registration/TrialReview.aspx?id=367612&isReview=true  https://www.publish.csiro.au/sh/sh20156 |
| IN-CA-164-1261 | PREPARATORY-5 | A pilot study of daily TDF/FTC-based PrEP among high-risk Toronto MSM:The PREPARATORY-5 Study | 52 | 52 | 0 | CAN (N=52) | M | 22–59 | 0 | NA | 10/11/2014 | 09/06/2016 | https://clinicaltrials.gov/ct2/show/NCT02149888  https://www.ncbi.nlm.nih.gov/pmc/articles/PMC5830065/ |
| IN-US-276-0122 | Flash PrEP | Houston HIV pre-exposure prophylaxis demonstration project | 36 | 34 | 0 | USA (N=34) | F, M | 23–63 | 0 | NA | 04/10/2013 | 30/12/2016 | https://www.ncbi.nlm.nih.gov/pmc/articles/PMC5781758/ |
| IN-US-276-1262 | PrEPception | PrEPception: expanding assisted reproduction options for serodiscordant couples | 25 | 23 | 23 | USA (N=23) | F | 21–47 | 0 | NA | 07/08/2014 | 30/04/2017 | https://clinicaltrials.gov/ct2/show/NCT02233192  https://www.ncbi.nlm.nih.gov/pmc/articles/PMC5564004/ |
| IN-US-276-1295 | FIGHT PrEP | Pre-exposure prophylaxis with TDF/FTC to prevent HIV-1 acquisition in young men and transgender women of color who have sex with men | 50 | 49 | 0 | USA (N=49) | M, T | 18–29 | 0 | NA | 09/02/2015 | 01/07/2016 | https://clinicaltrials.gov/ct2/show/NCT02367807  https://journals.lww.com/jaids/Fulltext/2018/10010/Delivery_of_TDF_FTC_for_Pre_exposure_Prophylaxis.7.aspx |
| IN-US-276-1340 | Women's PrEP Demo | PrEP to prevent HIV acquisition in US Women: a demonstration project | 38 | 38 | 0 | USA (N=38) | F | 27–62 | 0 | NA | 28/02/2017 | 15/07/2019 | https://clinicaltrials.gov/ct2/show/NCT03058835 |
| IN-US-276-1926 | Puerto Rican PrEP | Evaluating the feasibility and acceptability of implementing a PrEP program in PR-CoNCRA | 53 | 53 | 0 | USA (N=53) | M | 19–55 | 0 | NA | 17/11/2016 | 06/01/2019 | https://clinicaltrials.gov/ct2/show/NCT03116932 |
| IN-US-276-2122 | ToT CCTG601 | HIV pre-exposure prophylaxis linkage and adherence in men who have sex with men following completion of a PrEP demonstration project | 119 | 119 | 0 | USA (N=119) | M | 22–67 | 0 | NA | 07/01/2014 | 26/08/2017 | https://www.ncbi.nlm.nih.gov/pmc/articles/PMC6612450/ |
| **Total** |  |  | **20,872** | **17,274** | **6,598** |  |  |  | **101** |  |  |  |  |

AEs, adverse events; AUT, Austria; BEL, Belgium; BEN, Benin; BWA, Botswana; BRA, Brazil; CAN, Canada; CHN, China; DEU, Germany; DNK, Denmark; ECU, Ecuador; ESP, Spain; F, female; FRA, France; FRX, bone fracture; GBR, United Kingdom of Great Britain and Northern Ireland; IRL, Ireland; ITA, Italy; KEN, Kenya; MWI, Malawi; M, male; NA, not applicable; NGA, Nigeria; NLD, Netherlands; NZL, New Zealand; O, other; PER, Peru; RAE, renal adverse event; SEN, Senegal; T, transgender; THA, Thailand; TZA, United Republic of Tanzania; UGA, Uganda; USA, United States of America; ZAF, South Africa; ZWE: Zimbabwe.

**Supplementary Table S2.** **Investigator-Reported Bone and Renal AEs from the 72 Studies.**

| **AE term** | **Total number reported** | **Notes** |
| --- | --- | --- |
| **Bone AEs** |  |  |
| Acute right inferior pubic rami fracture | 1 |  |
| Arm (select only if part of arm unknown) | 1 |  |
| Avulsion fracture of the cuboid, left | 1 | Excluded |
| Avulsion fracture, right thumb | 1 | Excluded |
| Broke his foot due to a fall | 1 | Excluded |
| Broke his hand at a fall | 1 | Excluded |
| Broken arm traumatic | 1 | Excluded |
| Broken jaw ‘following attack’ | 1 | Excluded |
| Broken left foot (traumatic) | 1 | Excluded |
| Broken right foot (closed non-displaced fracture of the fifth metatarsal) | 1 |  |
| Clavicle fracture (traumatic) | 1 | Excluded |
| Clavicular fracture | 1 |  |
| Closed right radial fracture | 1 |  |
| Double fracture and torn ligament of the right ankle (self-report) | 1 |  |
| Facial bones fracture | 1 |  |
| Forearm fracture | 2 |  |
| Fracture left third toe (traumatic) | 1 | Excluded |
| Fracture of base of first metacarpal bone, left | 1 |  |
| Fracture of fourth phalange in left foot | 1 |  |
| Fracture right leg | 1 |  |
| Fractured right fifth finger due to trauma | 1 | Excluded |
| Fractured shoulder (self-report) | 1 |  |
| Hairline rib fracture #6 l (stable) | 1 |  |
| Head (skull, jaw, face bones) | 1 |  |
| Left ankle fracture | 2 |  |
| Left femur fracture | 1 |  |
| Left fibula fracture | 1 |  |
| Maxillary bone fracture | 1 |  |
| Mildly displaced fracture of the right aspect of the mandible | 1 |  |
| Non-pathological non traumatic broken right ring finger | 1 |  |
| Rib fracture (3rd, 8th and 9th) | 1 |  |
| Right 10th rib | 1 |  |
| Right foot avulsion fracture | 1 | Excluded |
| Right foot fracture | 1 |  |
| Right radial fracture | 1 |  |
| Right rib fracture | 1 |  |
| Right rib fracture (2) status post motor vehicle accident | 1 | Excluded |
| Small bones of the wrist/hands | 2 |  |
| Stress fracture right knee | 1 |  |
| Tooth fracture (tooth #2) | 1 | Excluded |
| Trauma to left index & middle finger – symptomatic lacerations & fracture | 1 | Excluded |
| Traumatic fracture of left ankle | 1 | Excluded |
| Traumatic fracture of middle phalanx versus right hand | 1 | Excluded |
| Traumatic fracture of phalange of right third digit with swelling of corresponding pip joint | 1 | Excluded |
| Traumatic fracture of the head of radius | 1 | Excluded |
| Traumatic fracture of the left hand | 1 | Excluded |
| Traumatic rib fracture | 1 | Excluded |
| Traumatic stress fracture of hip | 1 | Excluded |
| **Renal AEs** |  |  |
| [*No AE term reported*] | 9 | Excluded |
| Acute kidney injury | 1 |  |
| Acute renal failure | 1 |  |
| Asymptomatic microscopic hematuria | 1 |  |
| Bilateral nephrolithiasis | 1 | Excluded |
| Blood creatinine increased | 11 |  |
| Burning during urination | 5 | Excluded |
| Creatinine clearance decreased compared to screening. | 1 |  |
| Creatinine decreased. creatinine clearance changed to grade 2 from normal | 1 |  |
| Creatinine elevation | 5 |  |
| Creatinine has decreased creatinine clearance has changed to grade 2 | 2 |  |
| Creatinine, high | 2 |  |
| Cyst of kidney | 1 | Excluded |
| Decreased creatinine clearance | 168 |  |
| Decreased creatinine clearance greater than 10% | 3 |  |
| Decreased creatinine clearance of greater than 10% | 1 |  |
| Difficulty urinating | 1 | Excluded |
| Dysuria | 15 | Excluded |
| Elevated creatinine | 195 |  |
| Elevated creatinine clearance | 61 |  |
| Elevated upcr | 1 |  |
| Fanconi-renal tubular toxicity secondary to TFV | 1 |  |
| Glycosuria | 4 |  |
| Grade 2 decrease creatinine clearance | 2 |  |
| Grade 2 decreased creatinine clearance | 4 |  |
| Haematuria | 2 |  |
| Hematuria | 2 |  |
| Increased micturition frequency | 1 |  |
| Increased urinary frequency | 1 |  |
| Irritable bladder | 1 | Excluded |
| Mycrohematuria | 1 |  |
| Nocturia | 2 |  |
| Nocturnal urinary frequency | 1 |  |
| Pollakiuria | 1 |  |
| Post-micturition dribbling | 1 | Excluded |
| Pre-renal failure+ | 1 |  |
| Problems urinating | 1 | Excluded |
| Proteinuria | 26 |  |
| Reduced creatinine clearance | 3 |  |
| Renal colic | 3 | Excluded |
| Serum creatinine elevated to >1.5 x baseline | 1 |  |
| Ureteric calculus in left ureter | 1 | Excluded |
| Urethral burning | 1 | Excluded |
| Urethral burning sensation | 1 | Excluded |
| Urethral discharge | 9 | Excluded |
| Urethral discomfort | 3 | Excluded |
| Urethral inflammation | 1 | Excluded |
| Urethral outflow unknown diagnosis | 1 | Excluded |
| Urethral sensibility | 1 | Excluded |
| Urinary frequency | 3 |  |
| Urinary retention | 1 | Excluded |
| Urinary urgency | 1 | Excluded |

AE, adverse event; TFV, tenofovir.

Listing of investigator-reported bone and renal AEs. Bone AEs that were reported to have resulted from trauma or those that likely resulted from a
traumatic injury were excluded from this analysis. Renal AEs with a likely etiology unrelated to TFV exposure and those without an AE term were also
excluded. AEs were adjudicated by R.J.L., M.D., and C.C.

**Supplementary Table S3.** **Resistance Analysis Results.**

| **Study ID/ Patient ID** | **FTC/TFV resistance-associated mutations** | **Non-polymorphic NNRTI resistance-associated mutations** | **Days PrEP start to diagnosis** | **Days PrEP end to diagnosis** | **Most recent TFV-DP measure, DBS (fmol/punch)** | **Average  TFV-DP measure, DBS (fmol/punch)** | **Most recent TFV measure, plasma  (ng/mL)** | **Average TFV measure, plasma  (ng/mL)** |
| --- | --- | --- | --- | --- | --- | --- | --- | --- |
| CO-US-164-0404 | None | None | 331 | 0 | BLQ | 25 | NA | NA |
| CO-US-164-0404 | None | None | 505 | 0 | 416 | 493.8 | NA | NA |
| CO-US-164-0404 | None | None | 56 | 22 | 25 | 25 | NA | NA |
| CO-US-164-0404 | None | None | 504 | 0 | BLQ | 23.7 | NA | NA |
| CO-US-164-0404 | None | None | 427 | 0 | 49.9 | 91.3 | NA | NA |
| CO-US-164-0404 | None | None | 422 | 0 | 77.2 | 179.3 | NA | NA |
| CO-US-164-0404 | None | E138A^a^ | 428 | 0 | BLQ | 25 | NA | NA |
| CO-US-164-0404 | None | None | 169 | 0 | BLQ | 24 | NA | NA |
| CO-US-164-0404 | K65R, K70R, M184IV | K103N | 359 | 0 | 25 | 48.7 | NA | NA |
| CO-US-164-0404 | None | K103N | 536 | 0 | BLQ | 25 | NA | NA |
| CO-US-164-0404 | None | None | 420 | 0 | 110 | 258.7 | NA | NA |
| CO-US-164-0404 | None | None | 247 | 0 | 219 | 340.1 | NA | NA |
| CO-US-164-0404 | M184IV | None | 333 | 0 | 160 | 155.5 | NA | NA |
| CO-US-164-0404 | None | None | 420 | –81 | BLQ | 25 | NA | NA |
| CO-US-164-0404 | K65R, K70R, M184IV | None | 482 | 0 | 44.5 | 260.4 | NA | NA |
| CO-US-164-0404 | None | None | 185 | 0 | BLQ | 27.8 | NA | NA |
| CO-US-164-0404 | None | None | 173 | 0 | 104 | 199.7 | NA | NA |
| CO-US-164-0404 | None | None | 272 | 0 | BLQ | 34 | NA | NA |
| CO-US-164-0404 | None | None | 254 | 0 | BLQ | 25.1 | NA | NA |
| CO-US-164-0404 | None | None | 256 | 0 | BLQ | 25 | NA | NA |
| CO-US-164-0404 | None | None | 414 | 0 | 50.8 | 46.9 | NA | NA |
| CO-US-164-0404 | None | None | 501 | 0 | 55.3 | 62.6 | NA | NA |
| CO-US-164-0432 | K65R, K70R, M184IV | K103N | 132 | 0 | 36.4 | 76.2 | NA | NA |
| CO-US-164-0441 | M184IV | None | 92 | –5 | NA | NA | NA | NA |
| CO-US-164-0441 | M184IV | None | 108 | 6 | NA | NA | NA | NA |
| CO-US-164-0441 | None | None | 58 | 0 | NA | NA | NA | NA |
| CO-US-164-0452 | None | None | 230 | 3 | 12.5 | 137.5 | NA | NA |
| CO-US-164-0452 | None | None | 28 | 24 | 12.5 | 12.5 | NA | NA |
| CO-US-164-0452 | None | None | 295 | 0 | 12.5 | 34.9 | NA | NA |
| CO-US-164-0454 | M184IV | None | 36 | 0 | NA | NA | NA | NA |
| CO-US-164-0455 | None | None | 250 | 31 | 25 | 122.2 | NA | NA |
| CO-US-164-0455 | None | None | 225 | 1 | 89.8 | 145.5 | NA | NA |
| CO-US-164-0468 | None | None | 236 | 0 | NA | NA | BLQ | 0.31 |
| CO-US-164-0468 | None | None | 83 | 0 | NA | NA | 1 | 1 |
| CO-US-164-0468 | M184IV | None | 168 | 0 | NA | NA | 54.5 | 47 |
| CO-US-164-0468 | K70R, M184IV, K219Q | Y181C | 168 | 0 | NA | NA | 124 | 124 |
| CO-US-164-0468 | M184IV | None | 28 | 0 | NA | NA | 47.5 | 47.5 |
| CO-US-164-0468 | M184IV | None | 27 | 0 | NA | NA | 74.6 | 74.6 |
| CO-US-164-0468 | None | E138A^a^ | 58 | 0 | NA | NA | 1 | 0.9 |
| CO-US-164-0468 | None | None | 168 | 0 | NA | NA | 1 | 1 |
| CO-US-164-0468 | None | None | 28 | 0 | NA | NA | 54.4 | 54.4 |
| CO-US-164-0468 | L210W, T215D | K103N | 316 | 0 | NA | NA | 1 | 1 |
| CO-US-164-0468 | M184IV | None | 82 | 0 | NA | NA | 78.4 | 78.4 |
| CO-US-164-0468 | None | None | 26 | 0 | NA | NA | 90 | 90 |
| CO-US-164-0468 | None | None | 382 | 0 | NA | NA | BLQ | 0.4 |
| CO-US-164-0483 | None | None | 354 | 1 | BLQ | 25 | NA | NA |
| CO-US-164-0483 | K65R | K103N | 277 | 31 | BLQ | 25 | NA | NA |
| CO-US-276-0108 | M41L, D67N, T215V, K219E | G190A | 483 | 0 | NA | NA | NA | NA |
| CO-US-276-0108 | M41L, D67N, T215V, K219E | G190A | 175 | 0 | 25 | 84.4 | NA | NA |
| CO-US-276-0108 | None | None | 266 | 0 | 25 | 25 | NA | NA |
| CO-US-276-0115 | M184IV, K219Q, D67N, T215S | None | 106 | 0 | NA | NA | NA | NA |
| CO-US-276-1264 | M184IV | None | 32 | –1 | NA | NA | NA | NA |
| CO-US-276-1639 | None | K101E, K103N, E138A^a^, G190A | 269 | –20 | 74 | 109.5 | NA | NA |
| CO-US-276-1639 | None | None | 154 | 0 | 559 | 559 | NA | NA |
| CO-US-276-1639 | None | None | 273 | 0 | 25 | 144 | NA | NA |
| CO-US-276-1639 | None | None | 72 | 0 | 25 | 25 | NA | NA |
| CO-US-276-1712 | None | None | 244 | 1 | 2,257.5 | 2,245.9 | NA | NA |
| CO-US-276-1712 | None | None | 99 | 0 | 50.1 | 50.1 | NA | NA |
| CO-US-276-1774 | None | None | 30 | 0 | NA | NA | NA | NA |
| CO-US-276-1774 | M184IV | None | 21 | 0 | NA | NA | NA | NA |
| CO-US-276-1774 | None | None | 30 | 0 | NA | NA | NA | NA |
| CO-US-276-1774 | M184IV | None | 28 | 0 | NA | NA | NA | NA |
| CO-US-276-1774 | None | None | 114 | 0 | NA | NA | NA | NA |
| CO-US-276-1774 | None | None | 162 | 1 | NA | NA | NA | NA |
| CO-US-276-1774 | None | K103N | 30 | 0 | NA | NA | NA | NA |
| CO-US-276-1774 | None | None | 201 | 32 | NA | NA | NA | NA |
| CO-US-276-1774 | None | None | 29 | 0 | NA | NA | NA | NA |
| CO-US-276-2061 | None | None | 186 | 0 | NA | NA | NA | NA |
| CO-US-276-2061 | None | K103N | 51 | 0 | NA | NA | NA | NA |
| CO-US-276-2061 | None | None | 84 | 0 | NA | NA | NA | NA |
| CO-US-276-4368 | M184IV | None | 182 | 0 | NA | NA | NA | NA |
| CO-US-412-2055 | M184IV, T215TI | None | 28 | –8 | 1145 | 1145 | NA | NA |
| CO-US-412-2055 | None | None | 505 | –4 | 25 | 139.1 | NA | NA |
| CO-US-412-2055 | M184IV | None | 84 | –5 | 725 | 764.5 | NA | NA |
| CO-US-412-2055 | M184IV | K103N | 28 | 0 | 755 | 755 | NA | NA |
| CO-US-412-2055 | None | None | 125 | 30 | 25 | 108.5 | NA | NA |
| CO-US-412-2055 | None | None | 227 | 19 | 92.7 | 107.4 | NA | NA |
| CO-US-412-2055 | M184IV | None | 35 | 0 | 905 | 905 | NA | NA |

BLQ, below level of quantitation; DBS, dried blood spots; FTC, emtricitabine; NA, not available; NNRTI, non-nucleoside reverse transcriptase inhibitor; PrEP, pre-exposure prophylaxis; TFV, tenofovir; TFV-DP, tenofovir diphosphate.

^a^E138A is polymorphic in some HIV-1 subtypes and thus may not reflect prior NNRTI exposure.

**Supplementary Table S4. HIV-1 Incidence Rates After up to 96 Weeks in 68 Post-Approval Studies of F/TDF for Daily Oral PrEP.**

|  | **HIV-1 Diagnosis** | | **No HIV-1 Diagnosis** | | **IR (95% CI) per 100 person-years** |
| --- | --- | --- | --- | --- | --- |
|  | **n** | **Mean person-years** | **n** | **Mean person-years** |  |
| **All (N=15,473)** | **95** | **0.61** | **15,378** | **0.79** | **0.78 (0.64, 0.95)** |
| **Age at PrEP initiation 14 to <18 years (n=368)** | **4** | **0.51** | **364** | **0.56** | **2.0 (0.73, 5.2)** |
| **Age at PrEP initiation 18 to <35 years (n=10,466)** | **83** | **0.60** | **10,383** | **0.73** | **1.1 (0.88, 1.4)** |
| **Age at PrEP initiation 35+ years (n=4,089)** | **7** | **0.91** | **4,082** | **0.89** | **0.19 (0.091, 0.40)** |
| **TFV-DP in DBS not collected** | **43** | **0.42** | **9,212** | **0.71** | **0.66 (0.49, 0.89)** |
| **TFV-DP (fmol/punch) in DBS available** | **52** | **0.77** | **6,166** | **0.92** | **0.91 (0.70, 1.20)** |
| **<350 (<2 doses/week)** | **44** | **0.82** | **1,272** | **0.83** | **4.0 (3.0, 5.4)** |
| **350 to <700 (2-3 doses/week)** | **2** | **0.90** | **901** | **0.89** | **0.25 (0.062, 1.0)** |
| **700 to <1250 (4-6 doses/week)** | **6** | **0.35** | **2,215** | **0.94** | **0.29 (0.13, 0.64)** |
| **≥1250 (≥7 doses/week)** | **0** | **0.00** | **1,778** | **0.96** | **--** |
| **Cisgender men (n=9,209)** | **59** | **0.70** | **9,150** | **0.88** | **0.73 (0.57, 0.947)** |
| **Cisgender women (n=5,443)** | **33** | **0.41** | **5,410** | **0.63** | **0.97 (0.69, 1.4)** |
| **Transgender women (n=400)** | **2** | **1.03** | **398** | **0.75** | **0.66 (0.17, 2.7)** |
| **Transgender (n=337)** | **1** | **1.30** | **336** | **1.03** | **0.29 (0.041, 2.1)** |

**DBS, dried blood spots; IR, incidence rate; TFV-DP, tenofovir diphosphate.**
